# Preoperative serum carcinoembryonic antigen, albumin and age are supplementary to UICC staging systems in predicting survival for colorectal cancer patients undergoing surgical treatment

**DOI:** 10.1186/1471-2407-9-288

**Published:** 2009-08-20

**Authors:** Li-Chu Sun, Koung-Shing Chu, Su-Chen Cheng, Chien-Yu Lu, Chao-Hung Kuo, Jan-Sing Hsieh, Ying-Ling Shih, Shun-Jen Chang, Jaw-Yuan Wang

**Affiliations:** 1 Nutrition Service Team, Kaohsiung Medical University Hospital, Kaohsiung Medical University, Kaohsiung, Taiwan, Republic of China; 2 Department of Nursing, Kaohsiung Medical University Hospital, Kaohsiung Medical University, Kaohsiung, Taiwan, Republic of China; 3 Faculty of Medicine, College of Medicine, Kaohsiung Medical University, Kaohsiung, Taiwan, Republic of China; 4 Department of Anesthesia, Kaohsiung Medical University Hospital, Kaohsiung Medical University, Kaohsiung, Taiwan, Republic of China; 5 Department of Internal Medicine, Kaohsiung Medical University Hospital, Kaohsiung Medical University, Kaohsiung, Taiwan, Republic of China; 6 Division of Internal Medicine, Kaohsiung Municipal Hsiao-Kang Hospital, Kaohsiung, Taiwan, Republic of China; 7 Department of Surgery, Kaohsiung Medical University Hospital, Kaohsiung Medical University, Kaohsiung, Taiwan, Republic of China; 8 Department of Public Health, Faculty of Medicine, College of Medicine, Kaohsiung Medical University, Kaohsiung, Taiwan, Republic of China; 9 Graduate Institute of Medicine, College of Medicine, Kaohsiung Medical University, Kaohsiung, Taiwan, Republic of China; 10 Graduate Institute of Medical Genetics, College of Medicine, Kaohsiung Medical University, Kaohsiung, Taiwan, Republic of China

## Abstract

**Background:**

The aim of this study was to determine influence of prognostic factors in addition to UICC staging systems, on cancer-specific and overall survival rates for patients with colorectal cancer (CRC) undergoing surgical treatment.

**Methods:**

Between January 1996 and December 2006, a total of 1367 CRC patients who underwent surgical treatment in Kaohsiung Medical University Hospital were analyzed. We retrospectively investigated clinicopathologic features of these patients. All patients were followed up intensively, and their outcomes were investigated completely.

**Results:**

Of 1367 CRC patients, there were seven hundred and fifty-seven males (55.4%) and 610 (44.6%) females. The median follow-up period was 60 months (range, 3–132 months). A multivariate analysis identified that low serum albumin level (*P *= 0.011), advanced UICC stage (*P *< 0.001), and high carcinoembryonic antigen (CEA) level (*P *< 0.001) were independent prognostic factors of cancer-specific survival. Meanwhile, a multivariate analysis showed age over 65 years (*P *< 0.001), advanced UICC stage (*P *< 0.001), and high CEA level (*P *< 0.001) were independent prognostic factors of overall survival. Furthermore, combination of UICC stage, serum CEA and albumin levels as predictors of cancer-specific survival showed that the poorer the prognostic factors involved, the poorer the cancer-specific survival rate. Likewise, combination of UICC stage, age and serum CEA level as predictors of overall survival showed that the poorer the prognostic factors involved, the poorer the overall survival rate. Of these prognostic factors, preoperative serum CEA level was the only significant prognostic factor for patients with stage II and III CRCs in both cancer-specific and overall survival categories.

**Conclusion:**

Preoperative serum albumin level, CEA level and age could prominently affect postoperative outcome of CRC patients undergoing surgical treatment. In addition to conventional UICC staging system, it might be imperative to take these additional characteristics of factors into account in CRC patients prior to surgical treatment.

## Background

Colorectal cancer (CRC) is the most common cancer and also the third leading cause of cancer death in Taiwan, and it is also a significant health problem. In Taiwan, it is estimated that approximately 10000 CRC patients were diagnosed, and over 4100 patients died of this disease in 2006 (http://www.doh.gov.tw/statistic/index.htm; accessed in December 2008). The prognosis of CRC patients is mainly dependent on several factors: pathological, clinical and biological. Although pathologic stage [International Union against Cancer (UICC) classification] is useful for predicting prognosis in CRC patients, it is difficult to accurately determine the stage prior to surgical treatment [1]. Furthermore, it is well known that patients with the same UICC stage colonic and rectal cancers display survival heterogeneity, with some patients exhibiting relatively short survival times. Accordingly, the identification of more promising prognostic factors that are indeed highly predictive of CRC patients undergoing surgical treatment is mandatory. To date, a number of studies have been extensively conducted to explore the role of prognostic factors for survival in patients with CRC. Of these parameters, age, serum albumin, histology, and carcinoembryonic antigen (CEA) levels have previously been demonstrated to be powerful prognostic indicators for CRC patients [2-9]. However, information of an overall view of these factors in combination is scant. Combining these important prognostic factors might be important to be auxiliary to the UICC staging system in preoperative accurate prediction of cancer-specific and overall survival rates for CRC patients more precisely. The aim of this study was to identify clinical or pathologic variables that could be used preoperatively to predict postoperative cancer-specific and overall survival rates of CRC patients more accurately, in addition to conventional UICC staging systems.

## Methods

### Patients

This retrospective cohort study included 1422 consecutive patients with histologically proven CRC who were receiving surgical treatment at the Department of Surgery, Kaohsiung Medical University Hospital. Patients of postoperative mortality that was defined as death within the first 30 days after operation (n = 23) and/or having an incomplete record of medical charts (n = 32) were excluded. A total remaining 1367 patients were enrolled into this study. The study was approved by the Institutional Hospital Board of the Kaohsiung Medical University Hospital. Patients' clinical outcomes and survival status were regularly followed up till 31 December 2007. Available variables included: age of onsets, sex, tumor location, preoperative serum albumin, serum CEA, and TNM/UICC classification defined according to the criteria of the American Joint Commission on Cancer/International Union against Cancer (AJCC/UICC) [1]. We dichotomized continuous variables into two categories for statistical analysis including age: those aged <65 years (n = 624) and those ≥65 years (n = 743); serum albumin level: < 3.5 gm/dl (n = 392) and those ≥3.5 gm/dl (n = 975); serum CEA level: <5 ng/ml (n = 724) and ≥5 ng/ml (n = 643). All patients were followed up until their death, and only patients who died of CRC were included in the cancer-specific death category. Cancer-specific survival was defined as the time elapsed between primary surgery and death from CRC. Overall survival was defined as the time elapsed between primary surgery and death from any cause.

### Statistical analysis

All data were statistically analyzed using the Statistical Package for the Social Sciences, version 12.0 (SPSS Inc., Chicago, IL, USA). For the univariate statistical analysis, Chi-square test was used where applicable. A Cox proportional hazards model with forward stepwise variable selection was used for multivariate testing of those factors found to be significant by univariate analysis (the inclusion factors were those with *P *value less than 0.05 by univariate analysis). Overall and cancer-specific survival rates were calculated by the Kaplan-Meier method, and the differences in survival rates were analyzed by the log-rank test. A *P *value less than 0.05 was considered to be statistically significant.

## Results

The clinical and pathologic data regarding 1367 CRC patients are summarized in Table 1. There were 757 (55.4%) males and 610 (44.6%) females. Nine hundred and twenty (67.3%) patients had carcinoma of the colon, and 447 (32.7%) had carcinoma of the rectum. The median age of these patients was 66 years with a range of 19 to 95 years. Six hundred and twenty-four (45.6%) cases were < 65 years old and seven hundred and forty-three cases were (54.4%) ≥ 65 years old. With regard to the histological type of these tumors, 183 (13.4%) were well differentiated carcinoma, 1066 (77.9%) were moderately differentiated carcinoma, and 118 (8.7%) were poorly differentiated carcinoma. When classified with the UICC staging system, there were 230 (16.8%) stage I patients, 506 (37.1%) stage II patients, 391 (28.6%) stage III patients, and 240 (17.5%) stage IV patients.

**Table 1:** Demographic data in 1367 colorectal cancer patients

Variable	Case No.	Percentage (%)
Age		
<65 years/≥65 years	624/743	45.6%/54.4%
Gender		
Male/Female	757/610	55.4%/44.6%
Tumor size		
≥5 cm/<5 cm	539/774	39.4%/60.6%
Tumor location		
Colon/Rectum	920/447	67.3%/32.7%
Histological type		
Well/Moderately/Poorly	183/1066/118	13.4%/77.9%/8.7%
UICC^a ^Stage		
I/II/III/IV	230/506/391/240	16.8%/37.1%/28.6%/17.5%
Tumor invasion		
T1/T2/T3/T4	70/229/989/80	5.1%/16.7%/72.4%/5.8%
Node metastases		
N0/N1/N2	832/343/192	60.8%/25.2%/14.0%
Serum Albumin level		
<3.5 gm/dl/≥3.5 gm/dl	392/975	28.7%/71.3%
Serum CEA^b^level		
≥5 ng/ml/<5 ng/ml	634/724	46.4%/53.6%

^a^International Union Against Cancer

^b^Carcinoembrynic antigen

**Figure 1:**
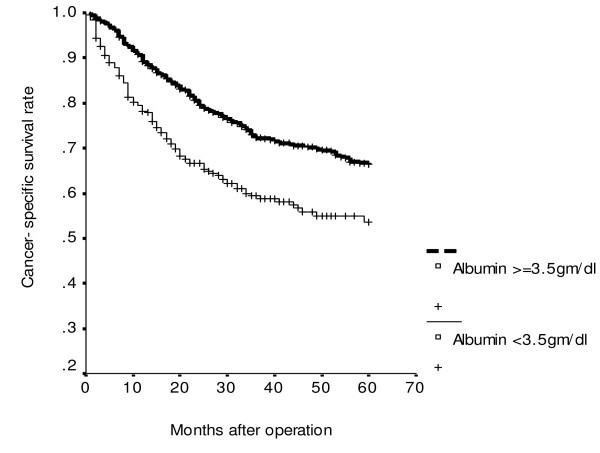
**Cumulative cancer-specific survival rates of patients with colorectal cancer according to serum albumin level (*P *< 0.001)**.

**Figure 2:**
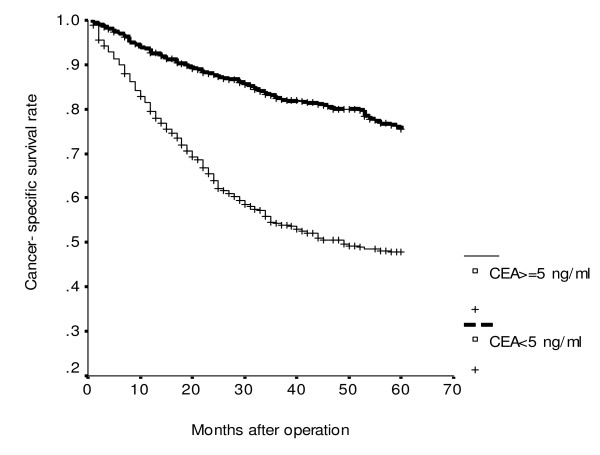
**Cumulative cancer-specific survival rates of patients with colorectal cancer according to serum carcinoembryonic antigen (CEA) level (*P *< 0.001)**.

**Figure 3:**
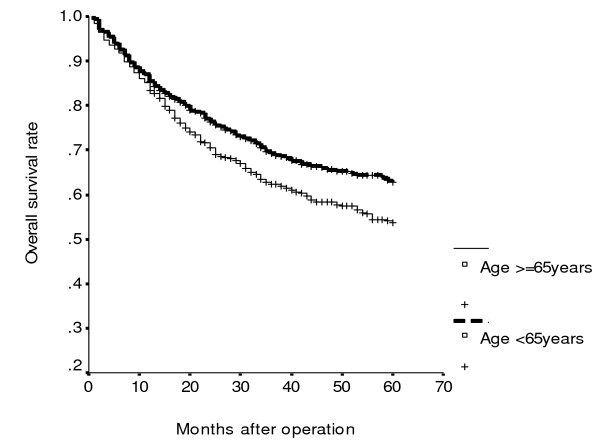
**Cumulative overall survival rates of patients with colorectal cancer according to age (*P *= 0.003)**.

**Figure 4:**
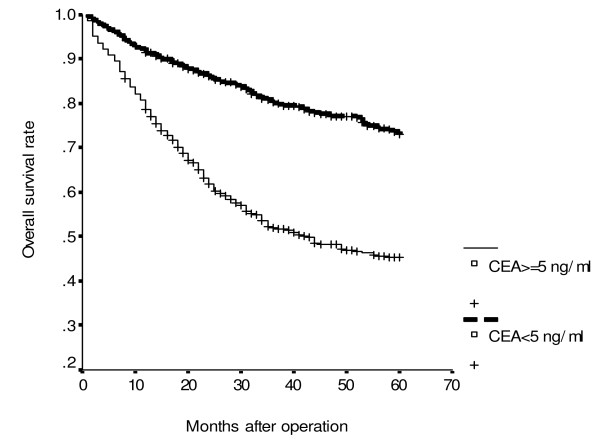
**Cumulative overall survival rates of patients with colorectal cancer according to serum carcinoembryonic antigen (CEA) level (*P *< 0.001)**.

Using univariate analysis of cancer-specific survival, we found that sex (*P *= 0.002), tumor size (*P *= 0.015), serum albumin level (*P *< 0.001), histology (*P *< 0.001), UICC stage (*P *< 0.001) and serum CEA level (*P *< 0.001) were statistically significant (Table 2). Moreover, Cox proportional hazards regression analysis indicated that patients with serum albumin levels < 3.5 gm/dl were 1.45 times more likely to die of cancer than those whose serum albumin levels ≥3.5 gm/dl (*P *= 0.011; HR, 1.25; 95% CI, 1.09–1.92); patients with UICC stage III/IV were 3.25 times more likely to die of cancer than those with UICC stage I/II (*P *< 0.001; HR, 3.25; 95% CI, 2.42–4.36); patients with serum CEA ≥5 ng/ml were 2.38 times more likely to die of cancer than those whose serum CEA <5 ng/ml (*P *< 0.001; HR, 2.38; 95% CI, 1.77–3.20) for cancer-specific survival. Moreover, the combination of UICC stage, serum CEA and serum albumin levels as predictors of cancer-specific survival is shown in Table 3. It was demonstrated, whatever the presence of any one predictor, or any two predictors or all three predictors, to be significant for cancer-specific survival of CRC patients (all *P *< 0.001). Meanwhile, the increased risk of cancer-specific survival is proportionate to the involved numbers of these three variables. CRC patients with serum CEA level < 5 ng/ml (*P *< 0.001; Figure 1) or albumin level ≥ 3.5 gm/dl (*P *< 0.001; Figure 2) had significantly greater cancer-specific survival rates than those with serum CEA levels ≥ 5 ng/ml or albumin level <3.5 gm/dl respectively. Moreover, CRC patients with age < 65 years (*P *< 0.001; Figure 3) or serum CEA levels < 5 ng/ml (*P *= 0.003; Figure 4) had significantly greater overall survival rates than those with age = 65 years or serum CEA levels ≥ 5 ng/ml respectively.

**Table 2:** Univariate and multivariate analysis of prognostic indicators on cancer-specific survival for colorectal cancer patients

Parameters	Number	Univariate analysis		Multivariate analysis	
		Hazard ratio (95% CI) *P *value		Hazard ratio (95% CI) *P *value	
Age (≥65/<65)years	743/624	1.17(0.97–1.41)	0.930	-	-
Sex (Male/Female)	757/610	1.24(1.03–1.50)	0.022	-	0.281
Site (Colon/Rectum)	920/447	1.19(0.98–1.46)	0.085	-	-
Tumor size (≥5/<5)cm	539/774	1.28(1.05–1.56)	0.015	-	0.411
BMI^a ^(≥24/≥18.5–24/<18.5)	434/516/417	0.84(0.64–1.09)	0.185	-	-
Albumin (<3.5/≥3.5)gm/dl	392/975	1.72(1.38–2.14)	<0.001	1.45(1.09–1.92)	0.011
Histology (PD/MD/WD^b^)	118/1066/183	3.05(1.95–4.77)	<0.001	-	0.341
UICC^c ^stage (III&IV/I&II)	630/737	3.96(3.22–4.87)	<0.001	3.25(2.42–4.36)	<0.001
CEA^d ^(≥5/<5) ng/ml	643/724	2.91(2.34–3.62)	<0.001	2.38(1.77–3.20)	<0.001

^a^Body mass index

^b^Poorly differentiated; Moderately differentiated; Well differentiated

^c^International Union Against Cancer

^d^Carcinoembrynic antigen

**Table 3:** Combination of carcinoembryonic antigen, albumin supplementary to UICC^a ^staging system as predictors of colorectal cancer for cancer specific survival by Cox regression analysis

Parameters	Regression coefficient	Standard error	Hazard ratio (95% CI)	*P *value
UICC^a ^stage (III&IV/I&II)	1.376	0.106	3.96(3.22–4.87)	<0.001
UICC^a ^Stage III&IV and serum CEA^b ^≥5 ng/ml	2.162	0.172	8.69(6.20–12.18)	<0.001
UICC^a ^Stage III&IV and serum CEA^b ^≥5 ng/ml and serum albumin < 3.5 gm/dl	2.794	0.264	16.347(9.735–27.450)	<0.001

^a^International Union Against Cancer

^b^Carcinoembrynic antigen

Table 4 shows the univariate and multivariate analysis of overall survival, age (*P *= 0.003), sex (*P *= 0.026), tumor size (*P *= 0.026), serum albumin level (*P *< 0.001), histology (*P *< 0.001), UICC stage (*P *< 0.001) and serum CEA level (*P *< 0.001) were significantly correlated to overall survival by univariate analysis. Furthermore, Cox proportional hazards regression analysis indicated that patients over 65 years of age were 1.85 times more likely to die of cancer than those under 65 years of age (*P *< 0.001; HR, 1.85; 95% CI, 1.41–2.43); patients with UICC stage III/IV were 3.09 times more likely to die of cancer than those with UICC stage I/II (*P *< 0.001; HR, 3.09; 95% CI, 2.34–4.07); patients with serum CEA ≥ 5 ng/ml were 2.28 times more likely to die of cancer than those whose serum CEA <5 ng/ml (*P *< 0.001; HR, 2.28; 95% CI, 1.73–3.01) for overall survival. Moreover, the combination of age, UICC stage, and serum CEA level as predictors of overall survival is shown in Table 5. It was demonstrated, whatever the presence of any one predictor, or any two predictors or all three predictors, to be significant for cancer-specific survival of CRC patients (all *P *< 0.001). Similarly, the increased risk of overall survival was proportionate to the involved numbers of these three variables.

**Table 4:** Univariate and multivariate analysis of prognostic indicators on overall survival for colorectal cancer patients

Parameters	Number	Univariate analysis		Multivariate analysis	
		Hazard ratio (95% CI) *P *value		Hazard ratio (95% CI) *P *value	
Age (≥65/<65) years	743/624	1.30(1.09–1.56)	0.004	1.85(1.41–2.43)	<0.001
Sex (Male/Female)	757/610	1.22(1.03–1.46)	0.026	-	0.186
Site (Colon/Rectum)	920/447	1.20(0.99–1.45)	0.066	-	-
Tumor size (≥5/<5) cm	593//774	1.24(1.03–1.50)	0.026	-	0.256
BMI^a ^(≥24/<18.5–24/<18.5)	434/516/417	0.84(0.66–1.09)	0.186	-	-
Albumin (<3.5/≥3.5) gm/dl	392/975	1.70(1.38–2.10)	<0.001	-	0.072
Histology (PD/MD/WD^b^)	118/1066/183	2.92(1.91–4.49)	<0.001	-	0.245
UICC^c ^stage (III&IV/I&II)	630/737	3.45(2.84–4.18)	<0.001	3.09(2.34–4.07)	<0.001
CEA^d^(≥5/<5) ng/ml	643/724	2.74(2.23–3.37)	<0.001	2.28(1.73–3.01)	<0.001

^a^Body mass index

^b^Poorly differentiated; Moderately differentiated; Well differentiated

^c^International Union Against Cancer

^d^Carcinoembrynic antigen

**Table 5:** Combination of carcinoembryonic antigen, age supplementary to UICC^a ^staging system as predictors of colorectal cancer for cancer overall survival by Cox regression analysis

Parameters	Regression coefficient	Standard error	Hazard ratio (95% CI)	*P *value
UICC^a ^stage (III&IV/I&II)	1.238	0.099	3.45(2.84–4.18)	<0.001
UICC^a ^Stage III&IV and serum CEA^b ^≥5 ng/ml	1.979	0.157	7.23(5.31–9.85)	<0.001
UICC^a ^Stage III&IV and serum CEA^b ^≥5 ng/ml and age ≥65 years	2.222	0.203	9.22(6.19–13.74)	<0.001

^a^International Union Against Cancer

^b^Carcinoembrynic antigen

To identify the promising prognostic factors of cancer-specific and overall survival rates in stage II or stage III CRC patients preoperatively, we further tried to analyze stage II and III CRC patients respectively. Of these factors, preoperative serum CEA level was the only significant prognostic factor for patients with stage II (Tables 6 and 7) and III (Tables 8 and 9) CRCs in both cancer-specific and overall survival categories (all *P *< 0.005), despite age was also one independent prognostic factor of overall survival in stage II and III CRC patients.

**Table 6:** Univariate and multivariate analysis of prognostic indicators on cancer-specific survival for stage II colorectal cancer patients

Parameters	Number	Univariate analysis		Multivariate analysis	
		Hazard ratio (95% CI) *P *value		Hazard ratio (95% CI) *P *value	
Age (≥65/<65)years	290/205	1.68(1.11–2.53)	0.014	-	0.190
Sex (Male/Female)	281/214	1.15(0.78–1.69)	0.492	-	-
Site (Colon/Rectum)	334/161	1.02(0.68–1.53)	0.940	-	-
Tumor size (≥5/<5)cm	253/242	1.12(0.76–1.66)	0.572	-	-
BMI^a ^(≥24/≥18.5–24/<18.5)	146/185/164	0.82(0.45–1.51)	0.527	-	-
Albumin (<3.5/≥3.5)gm/dl	169/326	1.86(1.17–2.97)	0.009	-	0.101
Histology (PD/MD/WD^b^)	48/367/80	2.59(0.96–6.95)	0.060	-	-
CEA^c ^(≥5/<5) ng/ml	214/281	2.14(1.37–3.34)	0.001	2.39(1.46–3.90)	<0.001

^a^Body mass index

^b^Poorly differentiated; Moderately differentiated; Well differentiated

^c^Carcinoembrynic antigen

**Table 7:** Univariate and multivariate analysis of prognostic indicators on overall survival for stage II colorectal cancer patients

Parameters	Number	Univariate analysis		Multivariate analysis	
		Hazard ratio (95% CI) *P *value		Hazard ratio (95% CI) *P *value	
Age (≥65/<65) years	290/205	1.97(1.33–2.91)	0.001	1.72(1.05–2.82)	0.003
Sex (Male/Female)	281/214	1.80(0.76–1.54)	0.670	-	-
Site (Colon/Rectum)	334/161	1.03(0.71–1.51)	0.864	-	-
Tumor size (≥5/<5) cm	253/242	1.04(0.72–1.49)	0.843	-	-
BMI^a ^(≥24/<18.5–24/<18.5)	146/185/164	0.94(0.54–1.63)	0.969	-	-
Albumin (<3.5/≥3.5) gm/dl	169/326	1.79(1.17–2.75)	0.008	-	0.138
Histology (PD/MD/WD^b^)	48/367/80	2.42(0.97–6.03)	0.057	-	-
CEA^c^(≥5/<5) ng/ml	214/281	2.08(1.39–3.13)	<0.001	2.17(1.39–3.40)	0.001

^a^Body mass index

^b^Poorly differentiated; Moderately differentiated; Well differentiated

^c^Carcinoembrynic antigen

**Table 8:** Univariate and multivariate analysis of prognostic indicators on cancer-specific survival for stage III colorectal cancer patients

Parameters	Number	Univariate analysis		Multivariate analysis	
		Hazard ratio (95% CI) *P *value		Hazard ratio (95% CI) *P *value	
Age (≥65/<65)years	189/193	1.33(0.90–1.96)	0.148	-	-
Sex (Male/Female)	198/184	0.74(0.52–1.05)	0.092	-	-
Site (Colon/Rectum)	263/119	0.96(0.66–1.40)	0.818	-	-
Tumor size (≥5/<5)cm	170/212	1.24(0.86–1.78)	0.252	-	-
BMI^a ^(≥24/≥18.5–24/<18.5)	123/150/109	1.01(0.63–1.61)	0.984	-	-
Albumin (<3.5/≥3.5)gm/dl	110/272	1.35(0.85–2.15)	0.207	-	-
Histology (PD/MD/WD^b^)	62/278/42	2.11(0.91–4.92)	0.083	-	-
CEA^c ^(≥5/<5) ng/ml	193/189	1.74(1.22–2.50)	0.002	1.74(1.22–2.50)	0.002

^a^Body mass index

^b^Poorly differentiated; Moderately differentiated; Well differentiated

^c^Carcinoembrynic antigen

**Table 9:** Univariate and multivariate analysis of prognostic indicators on overall survival for stage III colorectal cancer patients

Parameters	Number	Univariate analysis		Multivariate analysis	
		Hazard ratio (95% CI) *P *value		Hazard ratio (95% CI) *P *value	
Age (≥65/<65) years	189/193	1.87(1.33–2.65)	<0.001	1.87(1.33–2.65)	<0.001
Sex (Male/Female)	198/184	1.38(0.96–2.00)	0.086	-	-
Site (Colon/Rectum)	263/119	1.01(0.70–1.46)	0.961	-	-
Tumor size (≥5/<5) cm	170/212	1.17(0.82–1.65)	0.393	-	-
BMI^a ^(≥24/<18.5–24/<18.5)	123/150/109	0.99(0.63–1.59)	0.987	-	-
Albumin (<3.5/≥3.5) gm/dl	110/272	1.38(0.89–2.14)	0.156	-	-
Histology (PD/MD/WD^b^)	62/278/42	2.15(0.97–4.74)	0.058	-	-
CEA^c^(≥5/<5) ng/ml	193/189	1.43(1.02–2.02)	0.039	1.43(1.02–2.02)	0.039

^a^Body mass index

^b^Poorly differentiated; Moderately differentiated; Well differentiated

^c^Carcinoembrynic antigen

## Discussion

This current study revealed the association of preoperative serum albumin level, CEA level and age with survival of CRC patients undergoing surgical treatment, which is adjuvant to conventional UICC staging system. Particularly, our study demonstrated that combining preoperative serum albumin level, CEA level and UICC stage significantly affect the cancer-specific survival of patients with CRC postoperatively. Meanwhile, the combination of serum CEA level, age and UICC stage prominently affected the overall survival of CRC patients postoperatively. In contrast to UICC pathological stage, the upmost important meaning is that these three factors including serum albumin level, CEA level, and age could be available preoperatively.

Consistent with previous investigations [5,8,10,11], preoperative low serum albumin may be a vital indicator and may predict an unfavorable prognosis for CRC patients. Serum albumin level has been traditionally used as a biochemical marker of individual nutritional status. Hypoalbuminemia is associated with the presence of a systemic inflammatory response, weight loss and metastatic tumor volume [12,13]. Moreover, serum albumin measurement is part of the liver function test battery, and for most patients it is measured even before cancer is diagnosed and before the staging protocol or the treatment program. The potential advantage of serum albumin level as a preoperative prognostic factor in CRC patients is that it is inexpensive, reproducible and powerful. Our results demonstrated that low serum albumin was an independent significant prognostic factor, even after adjusting for potential confounding factors. The possible explanation for the association between low serum albumin and poor survival in CRC patients might be due to cancer cachexia [5]. Besides, low serum albumin level is indicative of an ongoing systemic response, which causes the loss of body weight and body protein [5,10].

Since the first description in 1965, CEA has remained the most regularly examined tumor marker [14]. CEA is a high-molecular weight glycoprotein in the immunoglobulin superfamily of molecules, that plays a pivotal role in such biological phenomena as adhesion, immunity or apoptosis of the tumor cells and assessment of sensitivity to anti-tumor agents [15,16]. High serum CEA has been shown to be associated with a number of malignancies, including those of colorectal, breast, pancreas and lung types. Previous studies have shown that preoperative high serum CEA is associated with a poor prognosis [2-7,17-21]. Our results demonstrated that high serum CEA was an independent significant prognostic factor of all CRC patients and stage II/III CRC patients, even after adjusting for potential confounding factors. We found that high serum CEA levels were associated with poor survival in CRC patients, and the possible reason might result from increased tumor volume leading to a higher incidence of postoperative metastasis. Dixon et al. have demonstrated that patients with high CEA and low albumin levels likely reflect some type of systemic compromise from an activation of a metabolically active tumor, exhibiting a significantly decreased survival time in CRC patients [5]. However, these presumptions await further investigation for confirmation. We are endeavoring to find more efficient ways of combining independent factors instead of using single independent factors alone to predict survival time. Our results also reveal that combining serum albumin level, serum CEA level and UICC stage could be more accurate to predict cancer-specific survival rates of CRC patients.

On the other hand, several studies have also shown that old age is an independent prognostic factor associated with poor prognosis in CRC patients [22-25]. The higher postoperative morbidity rate in the older age patient group is because of the significant enhancement in common postoperative complications. Consistent with our observation, Schiffmann et al. also revealed that the worse prognosis was in older CRC patients [24]. Older age may be associated with cardiovascular diseases or other medical illness [26], and with the significantly higher American Society of Anesthesiologists (ASA) classifications [27]; hence, older age is associated with poor overall survival, but not cancer-specific survival, in CRC patients. Actually, patient age has a decisive impact on the short-term postoperative outcome of patients undergoing surgery for CRC [28]. Ultimately, our current study suggests that cancer-specific and overall mortality should be considered separately in survival analysis of CRC patients.

## Conclusion

In conclusion, preoperative serum albumin level, CEA level and age could affect postoperative outcome of CRC patients undergoing surgical treatment. Of these factors, preoperative serum CEA level is the only significant prognostic factor for patients with stage II and III CRCs. Preoperatively, the identified prognostic factors supplementary to UICC staging system may be potentially useful to improve the prediction of cancer-specific survival and overall survival in CRC patients. However, it will be necessary to analyze clinical data from multiple institutions to find additional related variables in order to develop a more efficient and accurate way for predicting surgical outcome of CRC patients.

## Abbreviations

CRC: colorectal cancer; CEA: carcinoembryonic antigen; ASA: American Society of Anesthesiologists

## Competing interests

The authors declare that they have no competing interests.

## Authors' contributions

LCS analyzed the data and wrote the manuscript. KSC, SZZ, CYL, CHK, JSH and YLS made substantial contributions in data acquisition, statistical analyses and data interpretation, and helped in manuscript preparation. SJC and JYW participated in study design and coordination. All authors read and approved the final manuscript.

## Pre-publication history

The pre-publication history for this paper can be accessed here:

http://www.biomedcentral.com/1471-2407/9/288/prepub
